# *TNF-*α gene expression in relation to TNF-α level and bone mineral density in Polish patients with celiac disease

**DOI:** 10.3389/fendo.2025.1670590

**Published:** 2025-10-09

**Authors:** Kinga Skoracka, Szymon Hryhorowicz, Michał Michalak, Marta Kaczmarek-Ryś, Iga Dziechciowska, Agnieszka Dobrowolska, Iwona Krela-Kaźmierczak

**Affiliations:** ^1^ Department of Gastroenterology, Dietetics and Internal Diseases, Poznan University of Medical Sciences, Poznan, Poland; ^2^ Doctoral School, Poznan University of Medical Sciences, Poznan, Poland; ^3^ Institute of Human Genetics, Polish Academy of Sciences, Poznan, Poland; ^4^ Department of Computer Science and Statistics, Poznan University of Medical Sciences, Poznan, Poland; ^5^ University Center of Cancer Diagnostics, Poznan University of Medical Sciences, Poznan, Poland; ^6^ Laboratory of Nutrigenetics, Department of Gastroenterology, Dietetics and Internal Diseases, Poznan University of Medical Sciences, Poznan, Poland

**Keywords:** gene expression, TNF-α, celiac disease, bone mineral density, osteoporosis

## Abstract

**Background:**

Osteopenia and osteoporosis are common complications of celiac disease (CD).

**Objectives:**

Given the inflammatory background of bone disorders, this study aimed to assess the impact of *TNF-α* gene expression and its serum level on bone mineral density (BMD) in individuals with CD.

**Patients and methods:**

The study at the start included 70 adults: 51 women and 9 men with CD, and 10 healthy controls: 8 women, 2 men. In the first phase of the study, densitometric measurements of the lumbar spine (L1–L4) and femoral neck (FN) were performed using dual-energy x-ray absorptiometry (DXA) to identify patients with celiac disease and decreased BMD. These patients constituted Group A (CD with osteopenia/osteoporosis, n = 12). Group B (CD with normal BMD, n = 6), and Group C (healthy controls with normal BMD, n = 6) were matched for sex, age and body mass to patients in group A. In the second phase of the study, *TNF-α* gene expression was quantified using the real-time quantitative PCR (qRT-PCR) method, and serum TNF-α levels were measured with an enzyme-linked immunosorbent assay (ELISA). In third phase, statistical analyses were conducted to investigate the correlations between *TNF-α* expression and serum TNF-α levels with bone and anthropometric parameters.

**Results:**

No statistically significant differences in *TNF-α* gene expression or TNF-α serum levels were found between the groups. In Group A, *TNF-α* expression did not correlate with bone or anthropometric measures. However, in Group B, lower *TNF-α* expression correlated with higher BMI, FN BMD, L1–L4 BMD, and Z-scores. In contrast, healthy controls showed a positive correlation between *TNF-α* expression and BMD, T-score, and Z-score of the FN. There was also no statistically significant correlation between TNF-α cytokine concentration, and the parameters studied in groups A and B.

**Conclusions:**

In this study, TNF-*α*, both in terms of gene expression and serum level, does not appear to correlate with BMD status in individuals with CD and osteopenia/osteoporosis. Larger longitudinal studies integrating additional cytokines and signaling pathways are needed to clarify the role of TNF-α in bone loss in celiac disease.

## 1 Introduction

Decreased bone mineral density (BMD) is a common complication of celiac disease (CD). Generally, osteoporosis is reported to be less than 20% and osteopenia less than 40% in the CD population, although research results are often variable. Some data show that at diagnosis, low BMD can affect up to 70% of patients with CD, and it improves after the initiation of a gluten-free diet (1). The consequences of reduced BMD can be fractures and reduced quality of life (2). The pathogenesis of bone loss in CD has traditionally been attributed to impaired intestinal absorption of calcium and vitamin D. However, emerging research indicates that the etiology is more complex and likely involves immunological and genetic mechanisms. Identifying novel risk factors for osteopenia and osteoporosis in CD is therefore of critical importance.

According to the inflammatory theory of osteoporosis, both local and systemic inflammation contribute to bone mass deterioration in individuals with CD. Chronic elevation of pro-inflammatory cytokines, particularly tumor necrosis factor-alpha (TNF-α), has been implicated in the pathophysiology of bone resorption (3). TNF-α promotes osteoclastogenesis by upregulating the expression of receptor activator of nuclear factor kappa-B ligand (RANKL), a key mediator of osteoclast differentiation and activation (4). Additionally, TNF-α stimulates the production of other cytokines that synergistically enhance RANKL signaling and further accelerate bone resorption (5, 6). Furthermore, in patients with CD, the cytokine imbalance may impact bone metabolism by directly affecting osteoclastogenesis and osteoblast activity (7). Increasing evidence suggests that the *TNF-α* gene -308G>A (rs1800629) polymorphism is associated with an increased risk of osteoporosis and reduced BMD (8). A growing number of studies are emerging that discuss the relationship between BMD and inflammation in patients with CD (9). However, no studies to date have focused on the association between *TNF-α* gene expression and altered bone parameters in patients with celiac disease. Therefore, our study aimed to investigate the impact of *TNF-α* gene expression on bone mineral density in patients with celiac disease.

## 2 Patients and methods

### 2.1 Patients

The study initially comprised 60 adults, including 51 women and 9 men, all diagnosed with CD and treated in the outpatient clinic of the Department of Gastroenterology, Dietetics, and Internal Medicine at Poznan University of Medical Sciences, as well as a control group of 10 individuals (8 women and 2 men). All patients provided their written informed consent. The Bioethics Committee of Poznan University of Medical Sciences approved the study protocol, number 824/21. The study was conducted following the guidelines included in the Helsinki Declaration.

### 2.2 Methods

#### 2.2.1 Study design and participant classification

**Figure 1:**
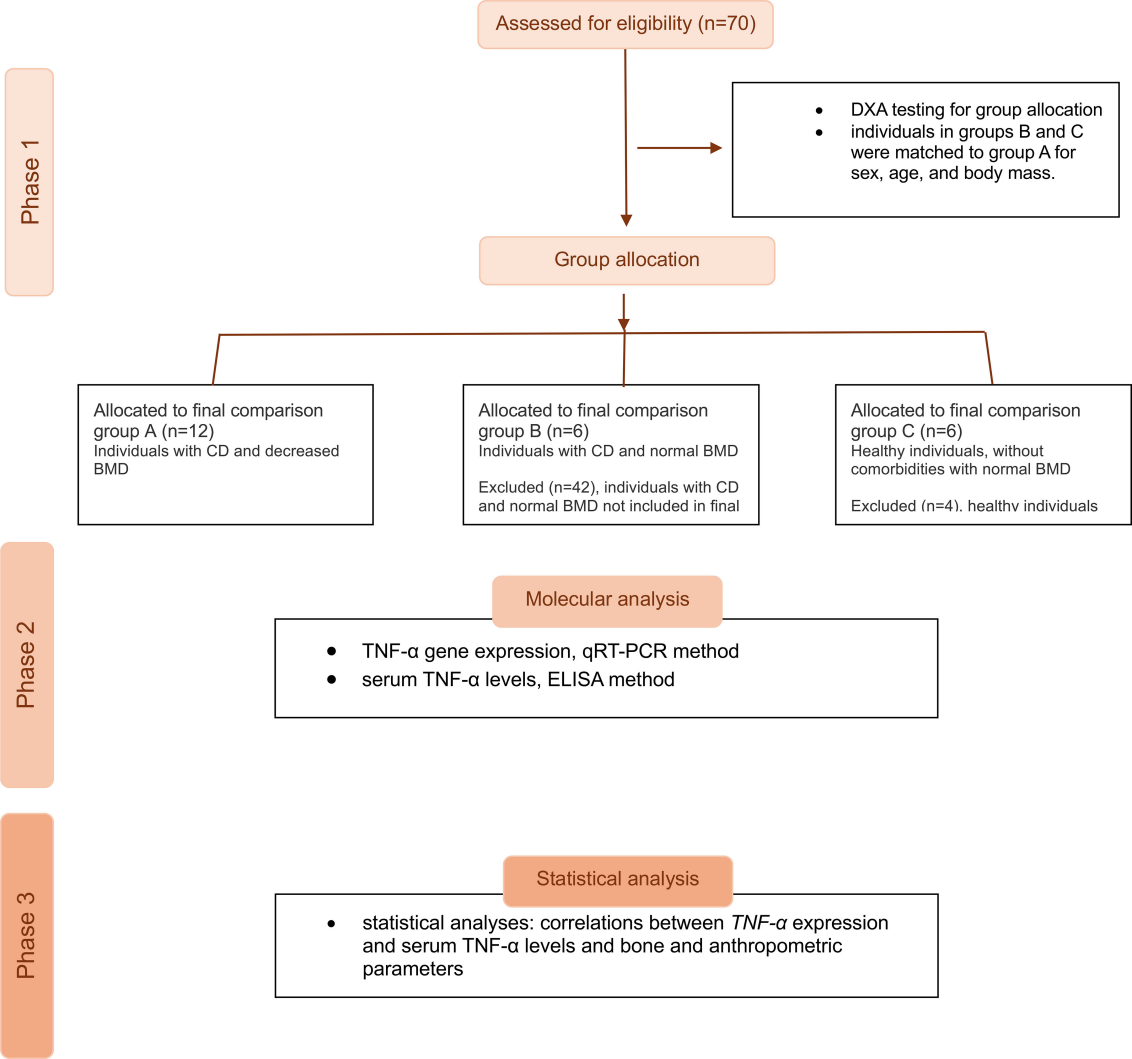
A flow diagram of the group allocation and phases of the study.

Densitometric examinations of the lumbar spine (L1-L4) and femoral neck (FN) were conducted on all study participants to evaluate BMD. Participants were categorised into three groups based on the T-score and Z-score values obtained. Further tests were then conducted within these groups to determine TNF-α concentration and assess *TNF-α* gene expression. Patients diagnosed with celiac disease and exhibiting reduced BMD were categorised into Group A (n = 12), while Group B comprised patients with CD and normal BMD (n = 6). Group C, serving as the control group, consisted of healthy subjects with normal BMD, without comorbidities (n = 6). Group B and C were matched for sex, age and body mass to patients in group A to ensure the groups were comparable **Figure 1** illustrates the workflow of the analysis.

#### 2.2.2 Diagnostic and exclusion criteria

The diagnosis of celiac disease was established based on clinical symptoms, serological testing (including tissue transglutaminase antibodies in the IgA class), and histopathological evaluation of small intestinal biopsies, following international guidelines. Participants were excluded if they were under 18 or over 50 years of age, or if they were postmenopausal women. Additional exclusion criteria included pregnancy, the presence of comorbid conditions known to affect BMD or nutritional status (such as inflammatory bowel diseases, malignancy, liver failure, chronic kidney disease, rheumatoid arthritis, or chronic obstructive pulmonary disease), the use of systemic glucocorticoids, or a lack of written informed consent to participate in the study.

All patients received standard clinical management based on the current recommendations of the European Society for Paediatric Gastroenterology, Hepatology and Nutrition (ESPGHAN) and the European Guidelines on Celiac Disease and Other Gluten-Related Disorders.

#### 2.2.3 Densitometric measurements

Bone mineral density was assessed at the L1–L4 and femoral neck using a Lunar DPX-Plus DXA scanner (Lunar, Inc., Madison, WI, USA). The measured parameters included the absolute BMD (g/cm²), the T-score, which reflects the deviation of the participant’s BMD from the mean BMD of a young adult population, and the Z-score, representing the age- and sex-adjusted deviation from the population mean. According to WHO guidelines, a T-score of −1.0 or higher is considered normal, a T-score between −1.0 and −2.5 indicates osteopenia, and a T-score of −2.5 or lower confirms a diagnosis of osteoporosis. Severe osteoporosis is defined by a T-score of −2.5 or lower in the presence of fragility fractures (10).

#### 2.2.4 RNA isolation and gene expression analysis

Peripheral blood samples (5 mL) were collected from each participant to enable total RNA extraction and subsequent gene expression analysis. RNA was extracted using TRIzol™ Reagent (Invitrogen, Waltham, MA, USA), followed by chloroform extraction, and then treated with DNase I (Bio-Rad Laboratories GmbH, Feldkirchen, Germany) to remove residual genomic DNA. The expression of the *TNF-α* gene was quantified using real-time quantitative PCR (qRT-PCR), with *GAPDH* (Glyceraldehyde-3-phosphate dehydrogenase) and *GUSB* (β-Glucuronidase) serving as internal reference genes. Primers were pre-validated and designed to generate amplicons not exceeding 250 base pairs, using commercially available assays Bio-Rad PrimePCR™ (Bio-Rad Laboratories GmbH, Feldkirchen, Germany). Before use, the primers were analyzed using NetPrimer software to ensure specificity and to identify any potential secondary structures, such as primer dimers or hairpin formations, that could interfere with amplification efficiency. Each qRT-PCR reaction was performed in three technical replicates, and the mean quantification cycle value (Cq) for *TNF-α* was calculated for each sample. The obtained Cq values were used for statistical comparison between the study groups, with lower Cq values indicating higher gene expression.

#### 2.2.5 Cytokine quantification

In addition to gene expression analysis, plasma concentrations of the TNF-α cytokine were measured in all collected blood samples using an enzyme-linked immunosorbent assay, following the manufacturer’s protocol (EIAab^®^ Science Inc., Wuhan, China kit, TECAN microplate reader, Tecan Group Ltd., Männedorf, Switzerland).

#### 2.2.6 Statistical analyses

Relative gene expression levels were quantified using the geNorm algorithm, which computes expression stability based on the geometric mean of the reference genes *GAPDH* and *GUSB*. Statistical comparisons of relative gene expression across experimental groups were conducted using one-way analysis of variance (ANOVA), followed by Tukey’s *post hoc* test for multiple comparisons, under the assumption that the variables follow the normal distribution. The comparison between two groups was performed by t-student test. The relationship between the *TNF-α* expression and analyzed parameters was using the Pearson’s correlation coefficient. All tests were considered significant at p< 0.05. The statistical analysis was performed using the statistical package Statistica 14 -Cloud Software Group, Inc. (2023) (Data Science Workbench, version 14. http://tibco.com).

## 3 Results

The study aimed to investigate the impact of *TNF-α* gene expression on the BMD of patients with CD. Moreover, we determined the serum concentration of *TNF-α*. Characteristics of the participants are presented in **Table 1**.

**Table 1:** Characteristics of celiac disease patients - group A and B - and the control group - group C.

Parameter	Group A	Group B	Group C	*p-value*
	Mean ± SD	Mean ± SD	Mean ± SD	
Age [years]	39.67 ± 7.11	39.83 ± 4.96	40.17 ± 7.89	0.898
Body mass [kg]	57.75 ± 17.19	60.83 ± 11.75	68.67 ± 3.33	0.305
BMI [kg/m^2^]	22.09 ± 4.75	21.47 ± 2.80	24.03 ± 2.75	0.498
BMD FN [g/cm^2^]	0.90 ± 0.17	1.00 ± 0.07	1.13 ± 0.08	0.006 0.005* 0.267** 0.221***
T-score FN	-1.01 ± 0.17	0.35 ± 1.02	0.73 ± 0.60	0.003 *0.006 **0.031 ***0.784
Z-score FN	-0.34 ± 1.08	0.90 ± 1.43	1.03 ± 0.68	0.027 *0.100 **0.148 ***0.976
BMD L1-L4 [g/cm^2^]	1.01± 0.07	1.21 ± 0.11	1.27 ± 0.06	<0.001 *<0.001 **0.001 ***0.405
T-score L1-L4	-1.51 ± 0.53	0.12 ± 0.91	0.70 ± 0.50	<0.001 *<0.001 **<0.001 ***0.271
Z-score L1-L4	-1.24 ± 0.75	0.45 ± 0.76	0.68 ± 0.53	<0.001 *<0.001 **0.001 ***0.837
*TNF-*α expression (Cq values)	34.40 ± 1.83	34.48 ± 1.27	35.26 ± 2.05	0.931
TNF-α [pg/ml]	5.29 ± 1.64	5.56 ± 1.62	5.81 ± 1.86	0.779

*p-value for differences between Group A and Group C; **p-value for differences between Group A and Group B; ***p-value for differences between Group B and Group C.

BMI, body mass index; BMD, bone mineral density; FN, femoral neck; L1–L4, lumbar spine; TNF-α, Tumor Necrosis Factor alpha; p < 0.05 was considered significant. Parameter values are presented as means and SD.

The analyzed groups were homogeneous in terms of age, body mass, and BMI, showing statistically significant differences between groups for bone parameters, including BMD, T-score, and Z-score in the FN and L1-L4 regions, which was expected given the study methodology.

We did not observe statistically significant differences between the groups in terms of *TNF-*α expression levels and TNF-α blood levels.

Nonetheless, we found differences in the mean values of *TNF-*α expression and *TNF-α* concentration between subjects with reduced BMD (group A) and those with normal BMD (groups B and C); however, these differences were not statistically significant. Detailed values are shown in **Table 2**.

**Table 2:** Mean Cq values for *TNF-α* gene expression and TNF-α protein levels in participants with reduced BMD (group A) and correct BMD (groups B and C).

Parameter	Group A (n=12)	Group B (n=6) and group C (n=6)	*p*-value
	Mean ± SD	Mean ± SD	
*TNF-*α gene expression (Cq)	34.40 ± 1.83	34.4 ± 1.36	0.764
TNF-α pg/ml	5.29 ± 1.64	5.66 ± 1.62	0.540

TNF-α, Tumor Necrosis Factor alpha; p < 0.05 was considered significant. Parameter values are presented as means and SD.

A correlation study was also performed to investigate the relationship between Cq values for *TNF-α* and bone and anthropometric parameters.

It showed that in patients with CD and osteoporosis (group A) there was no statistically significant relation between Cq values for *TNF-*α gene and the studied parameters. In patients with CD without osteoporosis (group B), a statistically significant, strong, positive correlation was observed between Cq values for *TNF-α* and the following values: BMI, BMD of the FN, BMD of L1-L4, and Z-score of L1-L4 (**Table 3**). In group C (healthy participants), a statistically significant, strong negative correlation was observed between the Cq values for *TNF-*α and the BMD, T-score, and Z-score of the FN.

**Table 3:** Comparison of correlations between Cq values for *TNF-*α and bone and anthropometric parameters.

Group	Parameter	(Cq)		
		R (X,Y)	r2	*p-value*
Group A	BMD FN [g/cm^2^]	0.42	0.18	0.170
	T-score FN	0.41	0.17	0.182
	Z-score FN	0.40	0.16	0.197
	BMD L1-L4 [g/cm^2^]	0.49	0.24	0.107
	T-score L1-L4	0.52	0.27	0.085
	Z-score L1-L4	0.34	0.12	0.282
	Body mass [kg]	0.33	0.11	0.303
	BMI [kg/m^2^]	0.49	0.24	0.105
	Anti-tTG [RU/ml]	-0.06	<0.01	0.882
Group B	BMD FN	0.99	0.97	<0.001
	T-score FN	0.36	0.13	0.481
	Z-score FN	0.26	0.07	0.618
	BDM L1-L4 [g/cm^2^]	0.83	0.68	0.043
	T-score L1-L4	0.78	0.60	0.070
	Z-score L1-L4	0.86	0.73	0.030
	Body mass [kg]	0.52	0.27	0.287
	BMI [kg/m^2^]	0.86	0.73	0.029
	Anti-tTG [RU/ml	-0.58	0.33	0.310
Group C	BMD FN [g/cm^2^]	-0.90	0.80	0.016
	T-score FN	-0.81	0.66	0.049
	Z-score FN	-0.84	0.70	0.037
	BDM L1-L4 [g/cm^2^]	-0.66	0.44	0.152
	T-score L1-L4	-0.80	0.64	0.057
	Z-score L1-L4	-0.64	0.41	0.173
	Body mass [kg]	0.51	0.26	0.301
	BMI [kg/m^2^]	0.12	0.01	0.822

BMI, body mass index; BMD, bone mineral density; FN, femoral neck; L1–L4, lumbar spine; TNF-α, Tumor Necrosis Factor alpha; Anti-tTG, anti-tissue transglutaminase; R(X,Y), correlation coefficient; r2, coefficient of determination.

There was also no statistically significant correlation between TNF-α cytokine concentration, and the parameters studied in groups A and B. In contrast, a strong positive correlation was observed in group C between TNF-α cytokine levels and BMI. The data are presented in **Tables 3** and **4**.

**Table 4:** Comparison of correlations between serum *TNF-α* concentration and bone and anthropometric parameters.

Group	Parameter	TNF-α serum concentration		
		R (X,Y)	r2	p
Group A	BMD FN [g/cm^2^]	0.17	0.03	0.666
	T-score FN	0.18	0.03	0.644
	Z-score FN	0.17	0.03	0.670
	BDM L1-L4 [g/cm^2^]	0.32	0.10	0.409
	T-score L1-L4	0.36	0.13	0.337
	Z-score L1-L4	0.07	0.01	0.856
	Body mass [kg]	0.29	0.08	0.453
	BMI [kg/m^2^]	0.60	0.36	0.090
	Anti-tTG [RU/ml]	-0.13	0.02	0.761
Group B	BMD FN	0.47	0.22	0.352
	T-score FN	-0.11	0.01	0.833
	Z-score FN	-0.19	0.04	0.715
	BDM L1-L4 [g/cm^2^]	0.74	0.54	0.095
	T-score L1-L4	0.81	0.66	0.051
	Z-score L1-L4	0.67	0.45	0.143
	Body mass [kg]	0.35	0.12	0.503
	BMI [kg/m^2^]	0.68	0.46	0.140
	Anti-tTG [RU/ml]	0.47	0.22	0.424
Group C	BMD FN [g/cm^2^]	-0.69	0.47	0.312
	T-score FN	-0.50	0.25	0.499
	Z-score FN	-0.39	0.15	0.613
	BMD L1-L4 [g/cm^2^]	0.24	0.06	0.763
	T-score L1-L4	0.67	0.45	0.333
	Z-score L1-L4	-0.23	0.05	0.774
	Body mass [kg]	0.94	0.88	0.057
	BMI [kg/m^2^]	0.95	0.91	0.047

BMI, body mass index; BMD, bone mineral density; FN, femoral neck; L1–L4, lumbar spine; TNF-α, Tumor Necrosis Factor alpha; Anti-tTG, anti-tissue transglutaminase; R(X,Y), correlation coefficient; r2, coefficient of determination.

**Table 5** summarizes the patients’ results, including expression levels, blood serum cytokine levels, gender, age, BMI, and bone parameters (T-score at FN and L1-L4).

**Table 5:** Specific characteristics of patients concerning Cq values for *TNF-α* and TNF-α concentrations.

Group	Lp	*TNF-α* expression (Cq)	TNF-α pg/ml	Sex (1-w, 0-m)	Age	BMI	T-score FN	T-score L1-L4
A	1	38.03	9.54	1	49	29.5	-0.60	-1.20
	2	32.12	n.d.	1	44	23.4	-3.00	-2.10
	3	33.24	4.45	1	43	17.4	-2.50	-1.60
	4	35.56	n.d.	1	33	21.0	-1.40	-0.30
	5	34.23	4.76	1	42	22.3	-1.70	-2.20
	6	36.21	5.06	1	35	22.8	-0.20	-1.50
	7	33.32	4.15	1	40	13.7	-1.30	-1.80
	8	34.56	4.76	1	48	21.1	-0.70	-1.90
	9	35.14	4.61	1	35	18.3	-1.20	-0.90
	10	32.87	5.52	1	45	22.9	-0.30	-1.40
	11	35.68	4.76	0	38	31.1	1.20	-1.40
	12	31.78	n.d.	1	24	21.6	-0.40	-1.80
B	1	33.58	3.90	1	47	20.2	-0.60	-0.90
	2	36.33	5.84	0	43	25.3	0.00	1.30
	3	34.65	4.76	1	40	20.0	1.60	-0.30
	4	32.63	5.52	1	33	18.6	-0.70	-0.30
	5	34.56	4.76	1	40	20.0	1.60	-0.30
	6	35.14	8.55	1	36	24.7	0.20	1.20
C	1	3263	n.d.	1	39	22.4	1.30	1.60
	2	35.84	4.71	0	25	22.1	0.10	0.60
	3	35.65	8.55	1	42	28.9	0.10	0.70
	4	35.35	5.37	1	46	22.0	0.40	0.30
	5	35.16	4.61	1	46	23.1	1.20	0.20
	6	33.55	n.d.	1	43	25.7	1.30	0.80

BMI, body mass index; FN, femoral neck; L1–L4, lumbar spine; TNF-alpha, Tumor Necrosis Factor alpha.

## 4 Discussion

In our study, we did not observe that the expression of the *TNF-α* gene and the serum concentration of TNF-α itself differed significantly depending on the BMD of the study participants, which may suggest that the level of gene expression and the concentration of the protein itself are not differentiating factors in the context of the occurrence of osteopenia and osteoporosis.

We also did not observe a correlation between the Cq values for *TNF-α* gene and bone and anthropometric parameters in the group of patients with CD and osteoporosis (Group A), which may suggest that the level of expression and concentration of the cytokine TNF-α does not play a significant role in regulating bone mass in people with CD and osteopenia or osteoporosis. On the other hand, among patients with CD and normal BMD, we observed a direct proportional relationship between the Cq values for *TNF-α* gene and BMI, BMD of the FN, BMD of L1-L4, and Z-score of L1-L4, which means that the lower *TNF-α* expression, the highest values of bone parameters. On the contrary, in healthy patients, we observed an inversely proportional relationship between Cq values for *TNF-α* gene and BMD, T-score, and Z-score of the neck.

The concentration of the TNF-*α* cytokine correlated directly with BMI among healthy patients, which is in line with the assumption that higher BMI and higher visceral fat content may be associated with increased production of pro-inflammatory cytokines.

In recent years, numerous studies have highlighted the role of genetic and immunological factors, as well as lifestyle factors, in the pathogenesis of osteoporosis (11). Celiac disease is also a disease with a complex background - genetic, immunological, and environmental (12).

TNF-α is a pro-inflammatory cytokine encoded by the *TNF-α*, located in human chromosome 6p21.3 (13). It is produced by macrophages/monocytes in response to acute inflammation and appears to be involved in the development of autoimmune diseases, such as celiac disease (14). Importantly, TNF-alpha has been shown to affect bone mineral density and contribute to the development of osteoporosis. The polymorphism -308G>A in the promoter of the *TNF-α* is intensively studied as a potential screening and diagnostic biomarker of osteoporosis. Interestingly, despite the reported negative effect of TNF-*α* on bone mineral density, meta‐analysis of 15 studies including 5273 participants demonstrated that the mutant model (GA+AA) of *TNF-α* -308G>A was associated with decreased susceptibility to osteoporosis, and the AA genotype was associated with increased BMD of the lumbar spine (9). On the other hand, a meta-analysis of 11 studies, involving 1147 cases and 1774 controls, indicated that this variant was associated with an increased risk of celiac disease (15).

Furthermore, a study conducted on patients with osteoporotic low-impact fractures demonstrated significantly elevated serum concentrations of TNF receptor 1 (TNF-R1) and TNF receptor 2 (TNF-R2) in both male and female subjects with osteoporosis compared to healthy controls, suggesting a role of TNF receptors in bone resorption (16).

Our study is the first to evaluate *TNF-α* expression in people with osteoporosis and celiac disease. We decided to examine the level of gene expression, as it allows us to assess its actual activity in the body, rather than just a potential predisposition, as in the case of polymorphism. In addition, gene expression levels are influenced by environmental, hormonal, and epigenetic factors, which could help identify potential modifiable factors that may reduce the risk of developing osteoporosis in celiac patients.

It is worth citing here the study by Ralston et al., who investigated the expression of mRNA encoding cytokines (IL-1, IL-6, and TNF) in freshly isolated human bone biopsy samples from individuals with postmenopausal osteoporosis. There were significant differences in the frequency of expression of specific cytokines, especially IL-1α, IL-1β, and IL-6, which were expressed significantly more frequently in bone samples from postmenopausal women with osteoporotic fractures compared to postmenopausal women with normal bone density or those on hormone replacement therapy (HRT). In contrast, *TNF-α* was expressed in a similar proportion of patients with osteoporosis and normal body weight (63% vs. 60%), while only 10% of patients treated with HRT expressed *TNF-α*. TNF-beta was less frequently expressed in osteoporosis (18%) and was not detected at all in postmenopausal patients with normal bone mass or those treated with HRT; however, the difference was not statistically significant in this study. Cytokine expression in bone samples from young healthy subjects showed sex-specific differences in cytokine expression. In men, the pattern was similar to that observed in healthy postmenopausal patients. In young women, the expression patterns were like those in the HRT group (17).

The results of this study cannot be directly compared to our observations due to the differences in methodology and study groups. Still, it indicates no significant differences in the level of *TNF-α* expression between individuals with normal BMD and those with osteoporosis; however, such differences can be observed in the levels of IL-1 and IL-6.

*In vitro* study has demonstrated that cytokines possess specific properties that influence osteoclast formation, but these effects are only observed in the presence of sufficient RANKL levels. TNF-α stimulated osteoclastogenesis in the early stage of cell differentiation, while IL-1β strongly stimulated osteoclastogenesis regardless of the conditions, and IL-6 inhibited it in all phases (18). In the late phase of cell differentiation, the effect of stimulation of osteoclastogenesis by TNF-alpha may be limited or even inhibitory, depending on the specific cellular conditions. The findings from this *in vitro* study have provided valuable insights into our results.

In patients with celiac disease and osteopenia/osteoporosis (group A), the lack of correlation between TNF-α expression and the parameters under study may reflect advanced bone metabolism disorders with a complex, multifactorial basis, wherein potentially other factors may mask the effects of *TNF-α*. In the group of patients with celiac disease and normal bone mass (group B), there is expected inverse relationship between the level of *TNF*-*α* expression and bone parameters, which is consistent with the known pro-resorptive effect of *TNF*-*α* on bone. In group C, the positive correlation between *TNF*-*α* expression level and bone parameters may be related to BMI, since the concentration of TNF-α cytokine was positively associated with this parameter. However, it is worth noting that the correlation between *TNF*-*α* expression and BMI, although positive, was not statistically significant.

Although gene expression testing is more accurate in assessing current gene activity than gene polymorphism, this method also has some limitations that may have affected the results. The level of *TNF-α* expression does not always correspond to the concentration of TNF-α protein in the blood, it is also worth noting that *TNF-α* expression can dynamically change depending on external factors - so the level of expression shows the actual state at the time of the test, while the development of bone disorders in patients with celiac disease is a continuous process. It would be very valuable to assess the level of expression at multiple time points over a more extended period, which would allow us to evaluate how bone mass and gene expression levels change.

## 5 Conclusions

In this study, *TNF-α* expression and the concentration of TNF-α in the blood serum were not correlated with bone and anthropometric parameters in individuals with CD and osteopenia/osteoporosis. However, in participants with CD and normal BMD, a decrease in *TNF-α* expression was correlated with improvements in certain bone parameters. Given the dynamic and multifactorial nature of *TNF-α* expression, future longitudinal studies are warranted to assess temporal changes in gene expression and cytokine levels in relation to bone remodeling and mass loss, particularly in autoimmune-related osteoporosis. Moreover, integrating analyses of other cytokines (e.g., IL-1, IL-6) and signaling pathways (e.g., RANK/RANKL/OPG axis) may provide a more comprehensive understanding of the mechanisms underlying bone loss in celiac disease.

## Data Availability

The raw data supporting the conclusions of this article will be made available by the authors, without undue reservation.
